# A Blockchain Parallel Activity Architecture with Social Network Graphs as Carriers for Internet of Things Networks

**DOI:** 10.3390/s25041003

**Published:** 2025-02-08

**Authors:** Xin Cong, Lingling Zi

**Affiliations:** College of Computer and Information Science, Chongqing Normal University, Chongqing 401331, China; cx@cqnu.edu.cn

**Keywords:** IoT networks, blockchain transaction, parallel processing, social network graphs

## Abstract

There is a need for information transactions between nodes in the Internet of Things (IoT) and blockchain technology can guarantee the anonymity and security of such transactions. However, current blockchain systems require networks to be connected in real time, but IoT networks cannot fulfill this requirement. Therefore, we put forward a blockchain parallel activity architecture using carriers (CBPA), which is capable of deploying blockchain systems on IoT networks. Firstly, the blockchain operation architecture, and its components are demonstrated. Secondly, the generation methods of the carrier, the carrier block and the transaction block are designed, respectively. Additionally, a feature-to-transaction correspondence algorithm is proposed, with the objective of accommodating the previous work of nodes when they are in a disconnected state within the network. Thirdly, a parallel generation method for transaction blocks is designed to permit multiple nodes to collaborate in generating blocks, thereby reducing the difficulty of block generation while accelerating the generation speed. Finally, intra-block and cross-block conflict resolution algorithms, as well as a block consensus and fork processing algorithm, are designed to ensure that nodes can participate in blockchain activities without being at a disadvantage and obtain legal benefits even when operating on a network with high communication latency. Theoretical analysis indicates that CBPA has both security and liveness. The experimental results show that when the block size is 1MB, CBPA improves the average throughput by about 10% and reduces the average latency by about 14% compared to existing schemes. When the percentage of failed nodes reaches about 18%, the blocking time increases significantly, and the valid block rate decreases by 0.2%. The proposed CBPA architecture expands the applicable blockchain network and provides practical solutions for disconnected operations.

## 1. Introduction

In recent years, the research on Internet of Things (IoT) networks has made great progress and practical applications, but there are still inevitable problems [1,2]. For example, the anonymity between devices and people leads to insecure information data, unstable network connections, and low computing and storage capacity of handheld devices. Blockchain systems offer a practical solution to the aforementioned problems by giving nodes in the network the ability to determine whether information is secure or not. Existing research focuses on how to utilize blockchain to assist in solving the problems of IoT networks, such as attack and defense [3], distributed data storage [4], access control [5,6], and task scheduling [7]. However, there is a lack of research on how to deploy blockchain systems on IoT networks under non-real-time connection conditions. So, there is a need to study architectures and methods for deploying blockchain systems on IoT networks.

In blockchain systems, blockchain activities that nodes are involved in mainly include transaction generation, transaction verification, transaction packaging, block generation, block consensus, block appending, etc. In addition, nodes need to request the latest transaction and block information from neighboring nodes in a timely manner. The main motivation for nodes to participate in these activities is to obtain benefits, and the key is to gain an advantage in activities such as block generation and consensus, which is measured using timestamps. In other words, the earlier the generated block is broadcasted to the entire network, the easier it is to reach consensus, and the earlier the latest confirmed block is available, the better it is for mining new blocks. Under blockchain network conditions, early time means that the network transmission latency is low. This implies that the nodes in the network must be able to maintain a real-time connection. This is a requirement for the ability of current blockchain systems to collaborate with other fields for blockchain activities. In actuality, however, there is still a class of networks known as IoT that cannot meet the requirement for real-time connection.

Based on the analysis of the connection characteristics of the nodes in the IoT and their own capabilities, the deployment of blockchain systems on the IoT can meet practical needs like securely interacting information between anonymous nodes, obtaining benefits, and stimulating nodes to actively transmit beneficial information. Still, there are some problems to be solved, such as blockchain not being able to run in an intermittent network, low-capacity nodes not being able to participate in blockchain activities, etc. Therefore, this paper focuses on the deployment blockchain system for IoT, with the goal of conducting blockchain activities under a non-real-time network connection, as well as cooperative mining among nodes for mutual benefits. Compared to the existing research, the proposed architecture addresses the IoT-specific issue by combining blockchain technology with social network graphs. Additionally, it provides practical solutions for disconnected operation, a topic that has not received enough attention in previous research. To be specific, the key contributions of our paper are summarized below.

(1) In order to be able to operate blockchain systems on IoT networks, we construct a novel blockchain parallel activity architecture (CBPA) that addresses the challenges of blockchain deployment, including dynamic node joining and leaving, high transmission latency, and the inability to detect node behaviors.

(2) IoT nodes are at risk of network interruption or high transmission latency, which might cause them to lose the time advantage in the block generating process and be unable to receive transaction fees and block generation benefits. Therefore, we propose a block generation method using a social network graph as a carrier, including the generation of the carrier, carrier block, and transaction block, as well as the feature-to-transaction correspondence algorithm. This enables nodes to obtain legal benefits based on their previous work even if they leave the IoT.

(3) The PoW-based block generation method requires nodes with a large computing power, which contradicts the existence of many IoT nodes with low computing power. Therefore, we overcome the limitation that blocks can only be generated by a single node in current blockchain systems, and enable low computing power nodes to participate in block generation activities by reducing the difficulty of solving PoW. Simultaneously, we design a transaction block parallel generation method that allows multiple nodes to cooperate to generate the same block together, thereby improving transaction throughput and ensuring block security.

(4) In the process of nodes generating blocks in parallel, there are cases where the same feature hash of a carrier corresponds to multiple transactions, while multiple blocks are linked in the same parent block. Therefore, we design the intra-block conflict resolution algorithm and cross-block conflict resolution algorithm, respectively, to reduce the probability of blocks being discarded.

(5) IoT nodes may not be able to access all the blockchain transaction information, resulting in possible duplicate transactions, while high transmission latency makes blockchain forks possible. Therefore, we present a block consensus and fork processing algorithm to improve the security of the proposed CBPA architecture.

The remainder of this paper is organized as follows: Section 2 reviews the related work on IoT networks and blockchain technology. Section 3 presents the proposed blockchain parallel activity architecture. Section 4 describes the details of the proposed architecture, including the generation of carriers and carrier blocks, parallel generation of transaction blocks and conflict resolution, block consensus, and fork processing. Section 5 shows the theoretical analysis and performance evaluation. Finally, Section 6 summarizes the paper and discusses future directions.

## 2. Related Work

Aimed at constructing a blockchain activity architecture for IoT networks, this section introduces IoT and blockchain technologies.

**IoT networks.** Nodes participating in blockchain activities in current blockchain systems require the network to be in a real-time connected state. This allows for fast receipt of the last transaction and block information, as well as to quickly broadcast locally generated blocks to the entire network for consensus and subsequent competition for ledger rights. To the best of our knowledge, blockchain technologies and existing online payment methods (such as credit cards, Alipay, etc.) that depend on reliable third parties do not facilitate transactions when the network is in a non-real-time connected state. In recent years, researchers have paid attention to the combination of blockchain and IoT. Peng [8] et al. introduced a clustering-based collaborative storage scheme for blockchain in IoT networks. This scheme clusters storage-limited IoT nodes into multiple peers to update the entire blockchain. Bataineh et al. [9] utilized the Ethereum blockchain infrastructure to construct an IoT–blockchain integration architecture. This architecture divides the IoT nodes into rich clients and thin clients; however, only the rich clients could execute the mining process to obtain the benefits. Hu et al. [10] proposed a cross-chain mapping blockchain for scalable data management in massive IoT networks. The proposed blockchain utilizes available fog-cloud resources to improve off-chain IoT data storage scalability. Ganapathy et al. [11] proposed an attack detection model to distinguish between normal user behavior and adversary behavior in an IoT network. Wu et al. [12] designed a hierarchical Practical Byzantine Fault Tolerance (PBFT) consensus algorithm with dual blockchain, which is applicable to the residential IoT with a small scope, improving the efficiency of the consensus process, and reducing the overhead caused by the consensus process. To counter the Distributed Denial of Service (DDoS) attacks of IoT, researchers [13] integrated machine learning techniques with blockchain to enhance the detection and mitigation of DDoS threats. Deep et al. [14] utilized blockchain to assist in solving the problem of data protection and authentication of IoT devices. The key to this is the use of smart contracts to perform authentication, access control, and data exchange mechanisms for IoT devices. Some scholars have explored the integration of Artificial Intelligence (AI), blockchain, and IoT. For example, Zheng et al. [15] studied a wireless-powered IoT and they proposed a distributed multimode resource allocation algorithm to save energy consumption in transmitting state information. Saadat et al. [16] developed an intelligent algorithm to select the optimal number of mobile consensus nodes to minimize the negative impact of low-stability consensus nodes. Kumar et al. [17] integrated blockchain, authentication and key agreement mechanism, and explainable AI for securing smart consumer applications. Deebak et al. [18] proposed a blockchain-based remote mutual authentication scheme to provide security and privacy. The scheme could coexist with the IoT smart environment to decentralize user requests.

In summary, utilizing IoT networks and their intermittent connectivity devices, blockchain systems can be deployed to complete Local Area Network (LAN) data transactions when the network is disconnected, and blockchain public ledger appending and updating activities when the network is connected. However, the participation of nodes in these activities requires a lot of data exchange in both disconnected and connected states of the network. Compared to the existing architecture combining blockchain and IoT, this paper focuses on how to improve the transaction throughput under this architecture and how to motivate limited-capacity IoT nodes to participate in the IoT blockchain.

**Blockchain technologies.** Blockchain technology research has attracted widespread attention in both academia and industry. In academia, the chain-structured blockchain has withstood the test for more than a decade, while the Directed Acyclic Graph (DAG) structured blockchains represented by Obyte are currently undergoing practical validation. In industry, blockchain technology is being incorporated into a number of significant fields, including power and energy markets [19], IoT [20,21], and AI [22,23]. In the chain-structured blockchain system represented by Bitcoin [24], the goal of the miner nodes is to gain benefits, and the competition between nodes is the competition of computing power; in another chain-structured blockchain represented by Ethereum [25], the miner nodes are also in a competitive relationship with each other, but are competing for the ownership of the virtual currency. Furthermore, in the DAG-structured blockchain system [26,27,28,29], nodes need to validate two previous transactions before starting a new one, and conflict resolution relies on network-wide voting to achieve asynchronous consensus. In the absence of benefits and computing power, the security of such blockchain systems remains to be regularly confirmed. Fundamental blockchain technologies such as timestamps, smart contracts, and consensus algorithms enable trusted confirmation of transactions without the need to go through a separate centralized entity. Specifically, the use of encrypted timestamps allows for accessing and recording distributed digital accounts [30] and the built-in timestamp function records historical information about blockchain transactions, sends and verifies time-series-related information about transactions, and ensures that new records in the network have built-in audit trail information that can be appended and maintained [31]. Smart contracts are decentralized agreements using computer code that adheres to the rules of digital transactions and are triggered by time [32]. They contain rules and contractual terms for two or more parties and can automatically notify the relevant parties in the network when the terms are executed. Consensus algorithms ensure the proper functioning of the blockchain by verifying the legitimacy and correctness of transactions. In public blockchains, algorithms represented by PoW [33,34] and PoS [35,36] achieve anonymity and security, but have the negative impact of low throughput. In consortium blockchains, the Byzantine fault-tolerant family algorithm serves as the consensus core, which allows the blockchain to operate much more efficiently than public blockchains, but the performance decreases as the network grows larger [37,38,39].

In recent years, mining pools have been proposed to improve mining efficiency and stability by centralizing globally dispersed computing power. The advantage is that it lowers the threshold of computing power for nodes to participate in block-generating while obtaining more stable benefits; the disadvantage is the centralization of the blockchain network and the susceptibility to 51% attacks. Nodes must have a real-time connection network in order to access the last task and block information, as the mining pool operator specifies block generation. As a result, applying the mining pool straight to the IoT is not practical. The main differences between CBPA and the mining pool are as follows. (1) Parallelism. The parallelism of the mining pool is mainly reflected in the task allocation during the process of block generation, while the parallelism of CBPA is mainly reflected in the block generation where the transactions contained in the block are packaged by multiple nodes. (2) Network architecture. The mining pool tends to be centralized, while CBPA tends to be decentralized. (3) Security. The security of the mining pool is ensured by the computing power of the block, while the security of CBPA is ensured by the computing power of all the transactions contained in the block. (4) Network connection. Mining pools require nodes in the real-time connection network, whereas nodes in the CBPA allows an intermittent network connection.

To sum up, the current blockchain is mainly designed for real-time network connection scenarios. Our task is to allow nodes to participate in blockchain activities in non-real-time network connection scenarios. These existing methods cannot meet the application requirements, so new methods are urgently needed to accomplish this task.

## 3. The Proposed Blockchain Parallel Activity Architecture

The geographic location of certain IoT nodes varies greatly, such as moving cars, pedestrians using mobile phones, resulting in neighboring nodes that may be uncertain. Moreover, these nodes are anonymous and it is not possible to determine whether a node is malicious or not by its historical behavior. However, nodes in turn need to exchange information with neighboring nodes or other nodes in the network, e.g., traffic jam information between vehicles and life information between mobile phones. Therefore, by utilizing the inherent characteristics of blockchain, secure information exchange between anonymous nodes can be achieved and nodes can be incentivized to contribute trustworthy information on the basis of obtaining certain benefits. However, when deploying a blockchain system on the IoT, nodes with high transmission latency are usually at a disadvantage when participating in blockchain activities, severely reducing their possibility of obtaining benefits. Therefore, we consider in our architectural design that nodes collaborate with each other in activities such as blockchain generation and consensus, and allow nodes to receive benefits based on their contributions. We design carriers that can accommodate transactions to accomplish the goal that nodes can participate in blockchain activities even when a network outage occurs and can verify transactions in parallel to generate blocks. In this paper, we choose the social network graph as carriers and construct the blockchain architecture based on it.

**Figure 1:**
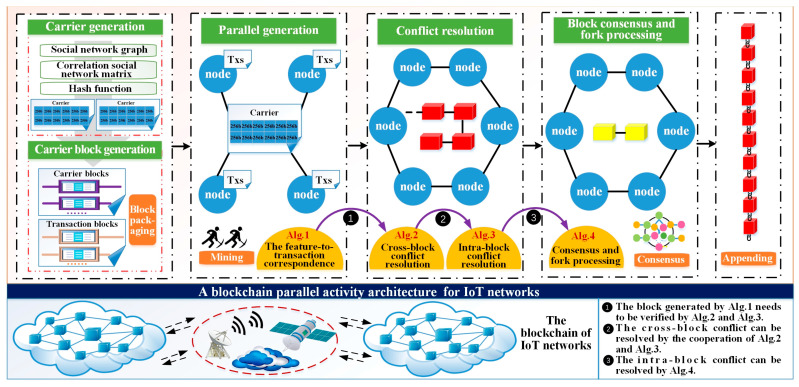
The proposed CBPA architecture for IoT networks.

The proposed CBPA architecture is demonstrated in Figure 1 and the node-based blockchain operation process can be briefly described as follows. First, the transaction initiator submits the transaction while generating the carrier using the social network graph, on which the carrier block is generated, and then the transaction and the carrier block are broadcasted throughout the network. Second, all nodes in the network choose one or more transaction and feature hashes from the received transaction and carrier blocks, and establish feature-to-transaction correspondence based on the mining difficulty and hash function. In this process, multiple nodes can work together to correspond different transactions to the same carrier and complete the parallel generation of transaction blocks. Third, nodes verify the generated blocks, eliminating those that are inconsistent with the mining difficulty and those that have conflicts both within and across transaction blocks. The verified blocks are then preformed for consensus across the network, and conflicting blocks are eliminated. Finally, the local blockchain is updated to complete the block appending. In the above operation process, the following problems need to be solved: (1) how to generate carriers that can accommodate transactions; (2) how to correspond the carrier feature hashes to the transactions; and (3) how to solve block conflicts in the block generation and consensus process. To address problem (1), we design carrier generation and carrier block generation methods, as detailed in Section 4.1 and Section 4.2. For problem (2), we design a parallel generation method (Algorithm 1), which is described in Section 4.3. For the block conflict in the generation process in problem (3), we design transaction block conflict resolution algorithms (Algorithms 2 and 3), which are described in Section 4.4. For the block conflict in the consensus process in problem (3), we design a block consensus and fork processing algorithm (Algorithm 4), which is described in Section 4.5. The proposed CBPA allows limited-capacity nodes to collaborate with each other in blockchain activities and ultimately obtain benefits both in case of single node or regional network disconnection and network connection. Compared to current blockchain systems, CBPA’s advantage is that it can support networks with high transmission latency, i.e., nodes can receive legal benefits based on their previous work even if they leave the blockchain system. Meanwhile, CBPA supports parallel block generation, which directly shortens the block generation time and improves the transaction throughput.

**Algorithm 1**: The feature-to-transaction correspondence algorithm**Input:** Transactions, carrier blocks and transaction blocks.**Output:** Corresponded transaction block. 1: Find the latest block in the locally stored blockchain and then parse the hash.2: Request the latest block from the neighbouring node and then parse the hash. 3: **if** (the two hashes are different) **then**4:  Update the locally stored blockchain and parse the hash of the latest block.5: **end if**//Preventing nodes from being attacked, reducing the probability of block generation being discarded, and getting the latest transaction block information6: Find the hash of the parent block in the locally stored transaction blocks.7: **if** (this hash is equal to the hash of the latest block) **then**8:  the node can select this carrier block for mining.9: **end if**//The purpose of 1–9 is to determine the carrier blocks that have not yet been corresponded10: Search for locally stored transaction blocks and count the number of correspond- ences between feature hashes and transaction blocks separately.11: Find transaction blocks with the maximum number for mining//The greater the number of features and transactions that already correspond, the earlier the block is broadcasted to the entire network for consensus, the higher the probability of it being received, and the higher the probability of gaining benefits.12: **if** (find multiple blocks) **then**13:   Select the transaction block with the earliest timestamp//Successful block consensus with high probability14: **end if/**/The purpose of 10–14 is to determine transaction blocks corresponding to existing partial transactions.15: Rank locally received transactions by transaction fees in descending order.//Getting higher transaction fee16:  Query feature hashes that have not been corresponded to transactions in the car rier blocks.17: Random numbers, timestamps and transaction data are used as inputs and PoW is used to compute the Merkel root and compare it with selected feature hashes until the set mining difficulty is satisfied.//Correspondence between features and transactions18: Fill in the transaction block.19: Broadcast the transaction block to the entire network after the transaction corresponds to the eigenvalue.//Completing the feature-to-transaction correspondence20: **return** the transaction block.

**Algorithm 2**: The cross-block conflict resolution algorithm**Input:** Transaction blocks to be verified broadcasted by other nodes**Output:** Legal transaction blocks1: The transaction blocks that are to be verified and that have been received over a period of time are entered into set *B*.2: **for** (transaction block *i* ∈ *B*) **do**3:  Check if transactions within *i* are legal//The same method as Bitcoin transaction checking.4:   Check if transactions within *i* already exist in another transaction block.//Avoiding repeat transactions.5:  Check if the difficulty of the feature-to-transaction correspondence within *i* is higher than or equal to the mining difficulty.//Avoiding reducing block security6:  Remove transaction blocks that do not pass 3–5 from *B*, indicating that they have been discarded.7: **end for**8: Find conflicting transaction block *B*’ with the same parent block hash from *B*.9: **for** (transaction block *i* ∈ *B*’) **do**10:  Check if the parent block is stored locally.//When the local block is incomplete, it may appear that the parent block is not found.11:  **if** (it is not) **then**12:   Request the parent block from neighboring nodes, remove *i* from *B*’ and store it locally for later verification.13:  **end if**14:  Count the computing power, transaction fees, timestamps of the transaction blocks that conflict with *i*.15:  Determine the unique transaction block *i*’ based on the principle of large computing power first, high transaction fee second, and early timestamp last.//Ensuring that the block is secure while protecting the benefits of the nodes as much as possible.16: **end for**
17: **return** *i*’

**Algorithm 3:** The intra-block conflict resolution algorithm**Input:** Conflicting transaction blocks.**Output:** Legal transaction block.1: Combine conflicting transactions corresponding to the same feature hash into set *B*.2: **for** (transaction *i* ∈ *B*) **do**3:  Calculate computing power of *i* and count transaction fee and timestamps of *i*.4: **end for**5: Find *i* with the largest computing power.//Ensuring block security6: **if** (find multiple largest computing power) **then**7:  Find the block *i* with the highest transaction fee.//Guaranteeing greater benefits for nodes8:  **if** (find multiple highest transaction fees) **then**9:   Find the block *i* with the earliest timestamp.10:  **end if**11: **end if**12: One-to-one correspondence between *i* and the feature hash. 13: **return** the legal transaction block.

**Algorithm 4:** The block consensus and fork processing algorithm**Input:** Transaction blocks with the same parent block**Output:** Transaction blocks appended to the blockchain1: Transaction blocks with the same parent block form a set *B*.2: **for** (transaction block *i* ∈ *B*) **do**3:  Check if there are any duplicate transactions within *i* and blocks with *i* as the parent block.//Preventing repeat transactions4:  Remove illegal *i* from *B* and also remove all transaction blocks with *i* as a parent block.5: **end for**6: **for** (*j* ∈ *B*) **do**7:  Calculate the number of transactions contained in *i* and all transaction blocks with *i* as a parent block.//Increasing transaction throughput8: **end for**
9: Find the transactions block *j* with the highest number of contained transactions.10: **if** (find more than one) **then**11:  Calculate the computing power contained in *j* and all transaction blocks with *j* as a parent block.12:  Find the transaction block *k* with the largest computing power.//Ensuring block security13:  **if** (find multiple blocks) **then**
14:   Find the transaction block *l* with the earliest timestamp.15:  **end if**
16: **end if**
17: Broadcast *l* and all transaction blocks with *l* as a parent block to other nodes.18: Consensus on *l* and all transaction blocks with *l* as the parent block using PBFT.19: **if** (the number of successfully verified messages received exceeds 2/3 of the number of nodes in the network) **then/**/the condition of PBFT successful consensus20:   **return** *l* and all transaction blocks with *l* as a parent block//transaction blocks that can be appended to the blockchain.21: **end if**

## 4. Methodology

In this section, we elaborate on the details of the CBPA architecture, including carrier generation (in Section 4.1) and carrier block generation (in Section 4.2), parallel block generating (in Section 4.3), intra-block and cross-block conflict resolution (in Section 4.4), and block consensus and fork processing (in Section 4.5).

### 4.1. Carrier Generation

In order to gain benefits, nodes in the current blockchain system need to be able to access the latest confirmed blocks and initiate transactions in a timely way, which requires the network to be able to connect in real time. When IoT nodes are in high transmission latency, it is difficult for nodes to obtain legal benefits. Meanwhile, under a normal network connection, these nodes consume a lot of computation, storage, and communication capabilities (expended costs) in block generation, block consensus, and message forwarding, and these expended costs are not rewarded once the network experiences high latency. Therefore, we design carriers that can carry the previous consumed costs of the nodes and the use a social network graph to generate the carriers.

Social network graphs are commonly used for modeling and analysis in the field of social networks. A social network graph usually consists of three parts: nodes, directed or undirected edges, and weights, and is stored and processed in the form of a matrix. Different social network graphs correspond to different matrices, and even if the number of nodes is the same, the correlation matrices are difficult to be cracked and forged due to the different names and ordering of the nodes, which provides a high level of security.

**Figure 2:**
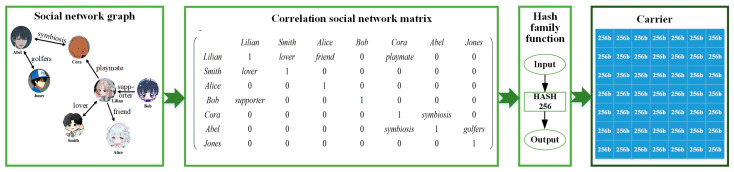
Illustration for the process of carrier generation.

Depending on their computing and storage capacity, nodes randomly generate social network graphs; that is, limited-capacity nodes choose simple random functions and nodes with high capabilities choose complex random functions. It should be mentioned that the social network graphs generated by different random functions have no impact on the carrier’s application, but the complex random functions increase the difficulty of cracking (refers to the behavior that carriers can still be appended to the blockchain after being tampered with by malicious nodes). Taking the randomly generated social network graph in Figure 2 as an example, different carrier generating nodes use different node names, number and direction of edges, and weights, which ensures that the social network graph cannot be known in advance. In addition, when generating the social network matrix, different ordering of each node name produces a different social network matrix, which ensures that even if the social network graph is obtained, it is impossible to fake the social network matrix. For example, using Hash256 as a hash function [40], taking (left node, right node, weight) as the input and finally generating a 256-bit string Hvalue, the above process is expressed as

(1) Hvalue=Hash256(left node⊙right node⊙weight)

Figure 2 illustrates the process of carrier generation. First, the nodes generate a social network graph using a random function, and then a certain ordering of the nodes is determined to obtain the correlation social network matrix. Second, the elements of each matrix are taken as inputs and computed by a pre-selected hash family function and the Merkel roots of the elements are derived using the form of a Merkel tree to obtain a hash with a fixed number of bits. This hash is filled in the appropriate position in the carrier matrix and is called the feature hash. The carrier-generating node ranks all the feature hashes, either by ranking the hash from lowest to highest or randomly, to form the carrier. It is worth noting that different ranking methods (i.e., different feature hash sequences) can also improve the security of the carriers.

Carrier generation relies on the social network matrix which is derived from the social network graph. The social network graph is generated locally by the carrier generation node with the following prerequisites. (1) Determining the number of transmissions that the carrier can accommodate. In general, a 1 MB-sized carrier block in the IoT can accommodate about 3000 transactions. (2) The social network matrix has the same number of feature hashes as the expected number of transactions, from which the size of the social network matrix is determined. (3) Nodes and edges matching the size of the social network matrix are generated locally using a random function and combined to form the social network graph. Thus, the process of generating the social network graph is conducted locally by the nodes, rather than by a network consisting of blockchain nodes, and the nodes do not need to be aware of the other nodes in the network.

The role of the carriers in the CBPA is as follows. (1) The carrier is a transaction block, but the generation of the block is parallel; i.e., the nodes complete feature-to-transaction correspondence at the same time. Compared with the limitation of the current blockchain that requires large computing power to generate a transaction block, feature-to-transaction correspondence can be accomplished with only a small amount of computing power, which enables limited-capacity nodes in the IoT to participate in the generation of the blocks as well. (2) In the current blockchain system, when the network is in a high-latency connection state, it means that locally generated blocks are not able to gain benefits with high probability. However, in the CBPA, by participating in block generation through carriers, the previous work is retained in the carriers, and the consensus is broadcasted to the entire network by the nodes with a normal network connection, so that all the nodes participating in the carrier generation can obtain legal benefits. Based on the above analysis, the use of carriers does not require any assumptions, i.e., there is no need to add additional devices to the current blockchain network or to change the data transmission protocol of the current blockchain. Furthermore, carrier generation based on social network graphs is appropriate for the following reasons. (1) The carrier is based on the social network, but the nodes locally generate the social network graph using the random function, so the cost of generating social network graphs is low. (2) The carrier carries about 2000~3000 transactions and needs about 2000~3000 feature hashes, and the social network graph, which can specify the number of nodes and edges, can fully satisfy this dynamic change in the number of transactions. (3) The source files of social network graphs can be stored in blocks in the form of a matrix, which serve as the basis for the verification and traceability of carrier feature hashes. (4) When calculating feature hashes, social network graphs have the advantages of fast calculation speed, low cost, and difficult to be cracked. In summary, comparing the existing media such as audio and video, only the social network graph meets the above requirements, so we use the social network graph as the basis for generating carriers.

### 4.2. Carrier Block Generation

**Figure 3:**
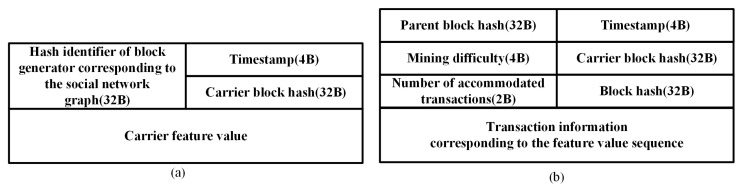
Illustration for the structure of blocks. (**a**) shows carrier blocks and (**b**) shows transaction blocks.

In the CBPA, the structure of carrier blocks and transaction blocks is shown in Figure 3. Carrier blocks are saved locally, while transaction blocks enable parallelization of blockchain activities. After calculating carrier feature hashes, the node fills the feature hash sequence into the carrier block in the format shown in Figure 3a. The structure of the carrier block consists of four items, the hash identifier of the block generator corresponding to the social network graph, the timestamp, the carrier block hash, and the carrier feature hash. Specifically, the hash identifier is the identity hash code of the carrier block generating node, which is the identification code assigned to the node when it first joins the blockchain system. It is unique and is used by that node to verify the authenticity of the carrier block in case the carrier block is forged. The timestamp is the time when that carrier block is generated, and the carrier block hash is the Merkel root of the binary storage file, which is determined after bits other than itself have been filled in, ensuring that the carrier block is not tampered with during propagation.

Figure 3b illustrates the structure of the transaction block, which consists of seven items. Specifically, the parent block hash is the hash of the previous block to which this block is linked in a chain-structured blockchain system. The carrier block hash and timestamp are the same as the corresponding items in Figure 3a. Mining difficulty is a value set by the block generator, in the same way that it is set in the Bitcoin system. That is, if the mining difficulty is high, the block that is eventually generated has an advantage in the overall network consensus, but transaction latency is long; if the mining difficulty is low, the block is generated quickly and transaction latency is short. The number of accommodated transactions is the number of transactions that each feature hash can accommodate. The transaction information corresponding to the feature hash sequence is a storage file of the transaction block, which corresponds to each feature hash one by one. After filling all the above information, the Merkel root of the transaction block is calculated and populated with the block hash to prevent the block from being tampered with.

Two problems need to be solved in the process of block packaging: one is how to select the feature hash and the other is how to select the transaction. For the first problem, the top-ranked feature hash has a higher probability of having a corresponding transaction, and if that feature hash is selected, it is prone to conflict and the corresponding block is more likely to be discarded. Therefore, it is better to find the unverified feature hashes in the ordering of the feature hashes based on the transaction blocks collected by the node for that carrier. For the second problem, selecting a transaction with a high transaction fee will obtain a large benefit; however, there is also a high probability that the transaction will be packaged by other nodes and that the block will be discarded, meaning that there will be no benefit. Thus, when the node’s computing power is large, transactions with high transaction fees are good choices, while when the node’s computing power is small, transactions with low transaction fees are more likely to be verified by repeated transactions.

When a node chooses a carrier block based on local collected transactions, it calculates the number of transactions to which the carrier’s feature hashes has been corresponded; the larger the number, the earlier the carrier becomes a carrier block and the more advantageous it is in the consensus process. The process of generating carrier blocks mainly includes the following steps.

Step 1. The nodes randomly generate a social network graph according to their capabilities and compute the correlation carrier feature hash matrix.

Step 2. The hash function is used on the elements of each feature hash matrix to compute their Merkel root, thus forming equal-length feature hashes.

Step 3. The ordering method is selected to rank the feature hashes, forming a storable binary sequence.

Step 4. Find the latest block on the blockchain based on the locally stored blockchain or by requesting the latest blockchain from a neighboring node.

Step 5. Set the mining difficulty and the number of accommodated transactions in the transaction block corresponding to the carrier feature hash.

Step 6. Fill in Figure 3a item by item to form the carrier block.

Step 7. Fill in Figure 3b item by item, where the block hash is the Merkel root after each item has been filled, forming the transaction block.

Step 8. Nodes broadcast carrier blocks and transaction blocks to the entire network, while keeping the social network graph, element ordering, and ranking rules locally stored for other nodes to check and retrieve during consensus.

### 4.3. Parallel Generation for Transaction Blocks

The node that initiates the transaction *T* generates a carrier block *B* at the same time, and then broadcasts *T* and *B* to the entire network, at which point other nodes can either correspond *T* to the feature hashes of the other carrier blocks or correspond other transactions to the feature hash of *B*. Depending on its computing power, a mining node may select generating multiple transaction blocks at the same moment, which may correspond to the feature hashes of multiple carrier blocks or to different feature hashes of a single carrier block. For the same carrier block, the accommodated transactions are jointly executed by multiple nodes and transaction fees are gathered collectively.

After receiving new transactions and carrier blocks, the mining node detects the transactions that have been corresponded in the carrier and counts the number of transactions whose feature hashes have been corresponded in the carrier. Subsequently, it selects the uncorresponded transactions and feature hashes, fills them into the transaction block, and broadcasts them to the entire network under the premise of satisfying the mining difficulty. The comprehensive procedure is delineated in Algorithm 1. The parallel generation process of transaction blocks shows that for a given transaction block, the filling work is conducted by multiple nodes in parallel, and for a given node, multiple transaction blocks can be selected to complete the filling work.

When the network is in a real-time connected state, IoT nodes have access to the latest blocks, transactions, and carrier blocks. In the case of a disconnected single-node network, a node can mine transaction blocks based on locally stored information and simultaneously mine multiple transaction blocks based on its computing power, and broadcast them to the entire network to reach a consensus once the network is connected. In the event that the regional network is disconnected, nodes and neighboring nodes can communicate through a network device, which cannot access the Internet but can form a LAN. Within this LAN, all blockchain activities described in Section 4.1, Section 4.2 and Section 4.3 can be operated without relying on the Internet.

In current blockchain systems, if a single-point network outage occurs, the mined blocks are discarded during the consensus process due to untimely broadcasting; if a regional network outage occurs, there is a significant latency in broadcasting it to the entire network, resulting in a long confirmation time. In the proposed CBPA architecture, a node completes only a part of the individual transaction block generation process, i.e., a feature-to-transaction correspondence. During a network disconnection at this node, the transaction blocks will be corresponded to by other nodes uninterruptedly, and will be broadcasted to the entire network after completing all correspondences. This process is independent of the nodes that are disconnected from the network; so, as long as a consensus is reached on the transaction blocks, the node can benefit from it even if a network disconnection occurs. In the case of a LAN disconnection, a node in the LAN initiates a transaction, which can then be received and verified by other nodes in the LAN as part of a single transaction block. This ultimately generates a single transaction block. Once the network is connected, the transaction block is broadcasted to the entire network in order to reach a consensus. Concurrently, the transaction generated by the node is also broadcasted to the entire network, at which point the other nodes in the network reach a consensus on the transaction block and verify the transaction. If the consensus is successful, even in the event of a network disconnection, the generated transactions can be confirmed in a shorter period of time.

### 4.4. Conflict Resolution for Transaction Blocks

The number of feature hashes is limited, and the number of nodes for mining the carrier blocks is uncertain. Due to network latency, nodes at different geographic locations receive the same message at different times, which could result in intra-block conflicts. That is, there are multiple transaction messages corresponding to the same feature hash and they all satisfy the mining difficulty, so then it is essential to uniquely determine the transaction when a conflict is detected in order to reduce the overhead of a subsequent resolution. Furthermore, it is possible that more than one transaction block may be linked to the same parent block, which could result in cross-block conflicts. Consequently, these blocks also need to be uniquely determined.

The metrics for resolving conflicts can be the computing power contained in the block, transaction fees, and timestamps. Specifically, the feature-to-transaction correspondence consumes the computing power of nodes, which needs to be consistent with the set mining difficulty. If the node executes the corresponding task with a difficulty higher than the mining difficulty, the security of the transaction block is enhanced. The larger the computing power contained in the block, the more difficult it is to be tampered with. Transaction fees are related to the benefit derived by the mining node; the higher the transaction fee, the greater the benefit of the node and the earlier the consensus time for that transaction block. The timestamp is related to transaction latency; the earlier the timestamp, the shorter the transaction latency. Based on the above analysis, Algorithm 2 provides details for resolving cross-block conflicts, while Algorithm 3 provides details for resolving intra-block conflicts.

In our blockchain architecture, nodes in the LAN are able to execute a transaction block conflict resolution algorithm without being connected to the Internet. They are then able to broadcast conflict-resolved transaction blocks to the LAN. Once the network is reconnected, the transaction blocks are broadcasted to the entire network so that the generated transaction blocks can reach consensus. Additionally, when some nodes that generate transaction blocks are experiencing network disconnection, the transaction block can also be broadcasted to the consensus of the entire network by the nodes that remain connected. Simultaneously, the block can be immediately broadcasted to the entire network, and nodes experiencing network disconnection can also obtain benefits.

### 4.5. Block Consensus and Fork Processing

After each feature hash in the transaction block corresponds one-to-one with the transaction, the transaction block hash is computed and filled in the transaction block. This transaction block is then broadcasted throughout the network for consensus. The consensus requires checking for duplicate transactions, conflicts within the same transaction block, and blockchain forks across the entire network. Duplicate transactions mean that during the process of Algorithm 3, the node determines whether a transaction is duplicated or not only by locally storing the blocks. In this case, when a node requests an update of the local blockchain from a neighboring node, it cannot acquire the latest block if it is in the LAN. Furthermore, the storage local blockchain is incomplete if the node has low capacity. Conflicts within the same transaction block refers to the fact that nodes with low capacity or network outages resolve conflicts within the transaction block based on locally stored blockchain information, and in the case where transactions may be missing, the final generated transaction block will be biased. A blockchain fork occurs when a transaction block is first generated, the transaction initiator selects the latest block on the blockchain as the parent block, and over time, there may be more than one block linked to the same parent block, or, due to network latency, the parent block that was chosen when generating the carrier block is no longer the latest block on the blockchain.

The key to resolving transaction conflicts is for nodes that are in the network-connected state to check whether the transaction has been verified and reached consensus. The solution for conflicts and forks within the same transaction block can be unified as determining the retained transaction blocks based on the number of transactions, computing power, and timestamps. Finally, consensus is reached through PBFT and the retained transaction blocks are appended to the blockchain. The detailed process is described in Algorithm 4.

In the CBPA, Algorithms 2–4 are required to check for duplicate transactions when resolving cross-block and inter-block conflicts, thus avoiding double-spend transactions as much as possible. Against Sybil attacks, a PBFT consensus strategy is used in the block consensus process, which is shown in reference [41] to prevent Sybil attacks.

## 5. Theoretical Analysis and Experimental Simulations

The proposed CBPA architecture with the goal of blockchain deployment on the IoT is evaluated from theoretical analysis and experimental simulations. For the theoretical analysis, we mainly prove its security and liveness. For the experimental simulations, we perform experiments to evaluate the performance such as transaction throughput, transaction latency, blocking time, and valid block rate.

### 5.1. Theoretical Analysis

In order to evaluate the performance of CBPA, it is analyzed in terms of both security and liveness. Security means that if two honest nodes submit transaction blocks with the same parent block hash, then the block hash of these two transaction blocks will be the same. Liveness means that the consensus protocol ensures that the node is able to generate the next block after generating the current transaction block. In other words, the node is able to receive the latest transaction block information in time. Assume *n* is the number of nodes, *f* is the number of fault-tolerant nodes, and then the number of honest nodes is 2*f* + 1. In this context, a fault-tolerant node refers to the maximum number of malicious nodes that can be accommodated; i.e., there are at most *f* nodes that either submit incorrect block information or do not submit block information.

**Security**. Suppose an honest node generates a transaction block *B* using Algorithm 3 and then broadcasts it to the entire network, reaching consensus with other nodes using Algorithm 4. If *h* is the block hash of *B*, then at least *f* + 1 honest nodes will receive *h*, and at most *f* fault-tolerant nodes will send a block hash *h*’ that is different from *h*. Consequently, this node can receive *f* + 1 verification pass messages for *B*; i.e., there is no honest node sending verification pass messages of *h*’ instead of *h*. When a generated block is converted from *B* to *B*’, *B*’ will have *B* as its parent block hash, which is determined by the chain structure. In order to be able to generate *B*’, at least *f* + 1 honest nodes need to be involved in the process of verifying *B*. Otherwise, *B* will be discarded due to not getting the required number of verifications. *B* then represents the last candidate block, influencing the generation of *B*’. From this, it can be concluded that the premise for generating each new block is to recognize the legitimacy of the last candidate block. This is to say that either *f* + 1 honest nodes confirm that block *B* is illegal, or *h*’ and *h* are consistent. However, for *h*’, since the number of verification pass messages does not meet the requirement, it will not be accepted by other nodes. Therefore, this inductive reasoning will always be applicable to the process of induction.

**Liveness**. In general, following the generation of a new block *B*, the receiving nodes undertake a verification process and subsequently broadcast the result. A node that behaves reliably (verifying the block and signing it) will receive a total of *f* + 1 verification pass messages. Conversely, a node that behaves unreliably (e.g., failing to forward verification messages or forwarding error verification messages) will eventually be identified and removed from the node’s neighborhood list according to the PBFT consensus protocol in Algorithm 4. When the number of fault-tolerant nodes reaches a threshold f (e.g., within a LAN in the IoT), the node will restart the block verification process, which serves to confirm the last parent block, transaction information, and so forth. Therefore, *B* is the last candidate block as long as the number of fault-tolerant nodes does not exceed *f*.

Furthermore, the number of times a node receives verification pass messages from *B* does not reach *f* + 1, the following four scenarios may be observed. In the first scenario, the parent block of *B* is the last block, thereby initiating the process of generating a new block. In the second scenario, *f* fault-tolerant nodes attempt to collaborate in order to make an error block the last candidate block. However, they are unsuccessful due to the absence of any verification pass messages from honest nodes. In the third scenario, the fault-tolerant node temporarily disguises itself as an honest node, thereby enabling *B* to become the last candidate block. In the final scenario, upon detecting a message exception (timeout), an honest node does not initiate a new block generation process. Instead, it awaits a verification pass message from *B*, while using Algorithm 1 to participate in the generation of other blocks. After detecting a malicious block verification message, the node discards the block and continues with the new block generation process. In accordance with the aforementioned scenarios, the last candidate block *B* will always be generated to ensure that the nodes are able to participate in the blockchain activities without any interruption.

### 5.2. Experimental Simulations

This section begins with an overview of the experimental environment, followed by a presentation of the baseline algorithms. Finally, the performance of CBPA is evaluated through a series of experiments.

#### 5.2.1. Experimental Environment

We collected real-world Ethereum TXs to build a dataset suitable for the IoT transaction architecture. There are 200,000 historical transactions in this dataset. We then chose 50 transaction blocks at random and paid close attention to the block ID, block hash, timestamp, and the number of TXs for each transaction. In our experiment, we calculated the decryption time of PoW puzzles and set the latency limit for this phase to 10 min. The consensus latency time of blocks is primarily attributable to the running PBFT protocol. Additionally, the Local Binary Pattern (LBP) approach was employed to extract feature hash from the social network graph. Each feature hash was then calculated using Hash256, which in turn generated the carrier blocks. It should be noted that the various extraction approaches have no impact on the performance of the proposed CBPA architecture.

We built a small-scale IoT system using Softether VPN 4.42 Build 9798 RTM. This system contains four servers, 10 routers, and 100 clients. The clients include 60 computers, 20 virtual machines installed in the servers, and 20 mobile devices such as mobile phones/tablets. Computers are used as normal nodes, and virtual machines and mobile devices are used as limited-capacity nodes. The main problems suffered by IoT nodes contain intermittent network connection, high transmission latency, high probability of packet loss, limited resources, and changes in neighboring nodes due to frequent joining and leaving. During the execution of experiments, these actual problems are simulated using the following way. (1) Modifying the router bandwidth profile to simulate the high transmission latency of the LAN in the IoT. (2) Modifying the bandwidth profile of virtual machines to simulate that different IoT nodes have different transmission rates. (3) Moving mobile devices to a geographic location with poor network signal to simulate high transmission latency of a single node in the IoT. (4) Mobile devices actively interrupt/resume the network to detect the impact of nodes dynamically leaving/joining the IoT on the entire blockchain architecture. To align with the demands of a real-world application, each node has a maximum bandwidth of 20 Mbps, with 10 outgoing and 100 incoming connections. When the nodes are in the network connected state, the average network latency is set to 32 ms (one-way transmission). When the nodes are in the network disconnected state, the average network latency in connecting to the Internet is set to 270 ms (one-way transmission), while the average network latency is set to 54 ms. Furthermore, the Transmission Control Protocol/Internet Protocol (TCP/IP) is employed for the transmission of data within a network. Additionally, the data structure of nodes in the CBPA primarily comprises their miner ID, neighbor node set, connected neighbor node set, available neighbor node set, current management node ID set, and IP address set.

#### 5.2.2. Baseline Algorithms

To evaluate the performance of the proposed CBPA, we compared two state-of-the-art baselines, namely Scheme 1 [42] and Scheme 2 [43].

Scheme 1: It proposes a digital payment scheme for providing blockchain services in remote areas, utilizing proxy nodes, distributed verification techniques, and probabilistic models to achieve delayed payments.

Scheme 2: It proposes a scheme for deploying a blockchain system in a telecommunications network, and uses the decentralized federated learning method deployed in the above scheme as an example to evaluate the effectiveness of the scheme from the perspectives of throughput and block confirmation latency metrics.

#### 5.2.3. Performance Evaluation

**Figure 4:**
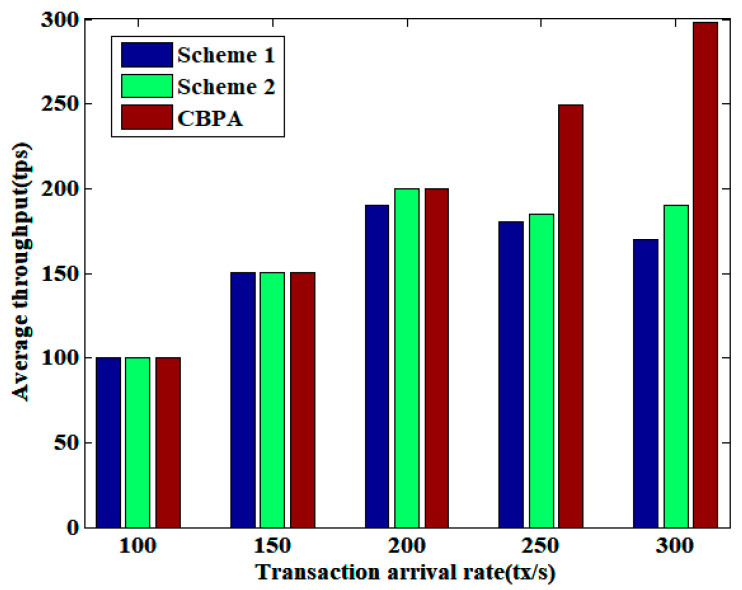
Comparison of average throughput under varying transaction arrival rates.

The average throughput of three schemes (including Scheme 1, Scheme 2 and the propose CBPA) under varying transaction arrival rates is compared in Figure 4. Transaction arrival rate is the number of transactions entering the blockchain network per second waiting for verification, while average throughput is the number of confirmed transactions per second. When there are fewer transactions waiting for confirmation in the blockchain network, the transaction throughput and arrival rate are balanced. However, when there are more transactions waiting for confirmation, for instance with an arrival rate of 250 or 300, the average throughput of the proposed CBPA is higher than that of the other two schemes. In addition, CBPA outperforms Bitcoin and Ethereum in terms of transaction throughput. Bitcoin generates a block in 10 min with an average throughput of 9tps, and Ethereum generates a block in 8 s with an average throughput of 23tps. This demonstrates the superiority of our proposed scheme, which is attributed to the parallel solution employed in the process of block generation, whereby nodes in the blockchain collaborate to generate blocks, thereby reducing the difficulty of block generation. The CBPA architecture minimizes wasted computing power compared to the single node generation of individual blocks, thus improving the speed of block generation.

**Figure 5:**
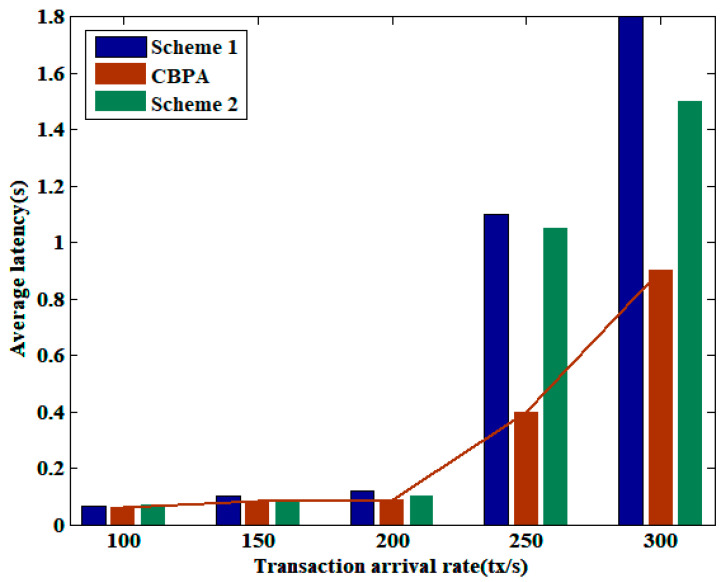
Comparison of average latency under varying transaction arrival rates.

Figure 5 presents a comparison of the transaction average latency of three schemes under varying transaction arrival rates, demonstrating how the average latency changes as the number of transactions to be confirmed in the blockchain network increases. As can be seen from the figure, there is an upper bound on the number of transactions that can be processed in a timely manner when the block size is fixed. When the transaction arrival rate exceeds a certain threshold, nodes are more inclined to select transactions with a high transaction fee when generating blocks, leading to an increase in transaction latency. Moreover, Scheme 2 requires multiple iterations during the optimization process and waits for nodes with low computing power to upload model parameters, resulting in a higher average latency than CBPA. In short, the parallelism of block generation and the liveness of the architecture provide CBPA with certain advantages in average latency.

**Figure 6:**
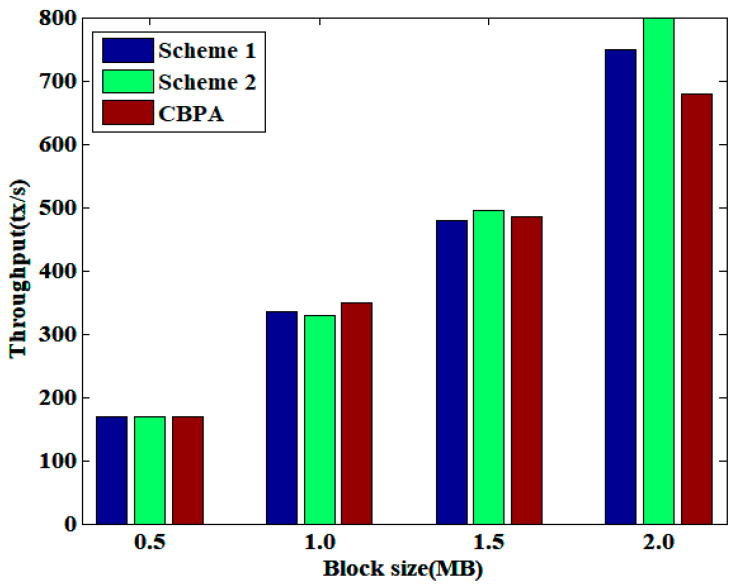
Comparison of transaction throughput under varying block sizes.

Figure 6 illustrates the changes in transaction throughput for block sizes of 0.5 MB, 1.0 MB, 1.5 MB, and 2.0 MB. From the figure, it can be seen that as the block size increases, the number of transactions that can be accommodated increases accordingly, thereby increasing transaction throughput. Nevertheless, an increase in block size results in an increase in the fork rate, which in turn prolongs the confirmation latency of certain transactions. At the same time, CBPA comprises nodes with high transmission latency, and when executing Algorithm 1, the probability of intra-block conflicts increases due to the delay in broadcasting the feature-to-transaction correspondence messages. This results in frequent execution of Algorithm 2 and a reduction in the speed of block generation. In the IoT, when the block size is large, the time spent on network transmission also increases accordingly, and there may even be transmission failures, resulting in a decrease in transaction throughput. So, compared to the other two schemes, CBPA exhibits lower transaction throughput for blocks with larger sizes.

**Figure 7:**
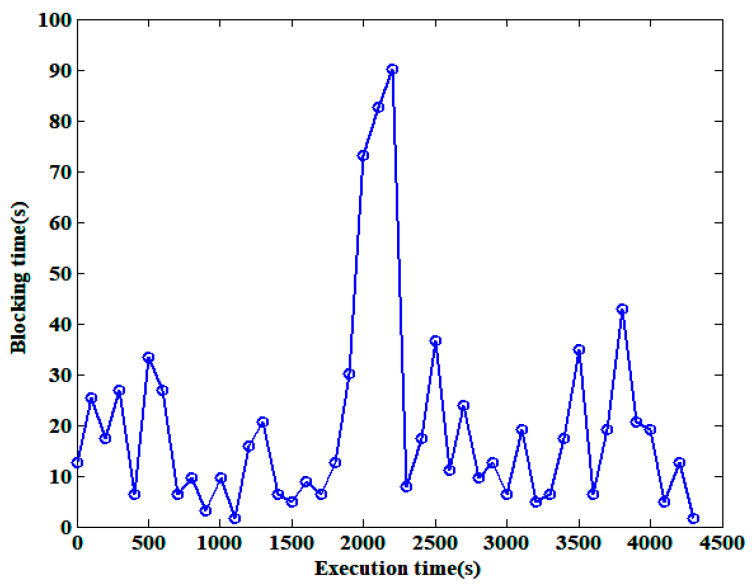
The impact of node failure on blocking time.

One of the main problems faced by IoT is node failure; i.e., a node fails to respond to a message before a set time threshold, thus affecting block generation and consensus processes. Figure 7 shows the time taken for blocking to occur for blocks with node failure rates between 5% and 20% when 50,000 transactions need to be confirmed. The horizontal axis represents the execution time of our method, and the vertical axis represents the blocking time. When the percentage of failed nodes reaches its maximum, there is a significant increase in the blocking time. According to Algorithm 1, the computation of feature-to-transaction correspondence is parallel, and the impact of node failure on block generation is limited. Moreover, nodes can obtain the current number of network nodes when generating carriers and then dynamically adjust the mining difficulty. However, due to the fixed mining difficulty of the blocks being generated when the node fails, the new mining difficulty will take effect with a delay in time. Algorithms 2 and 3 involve transaction conflict resolution, which requires nodes to reach a consensus. As the number of nodes responding in time decreases, the consensus time is extended. Subsequently, as the mining difficulty is adjusted and the total number of nodes in the network changes, the number of nodes that reach consensus also changes, resulting in a decrease and stabilization of the blocking time.

**Figure 8:**
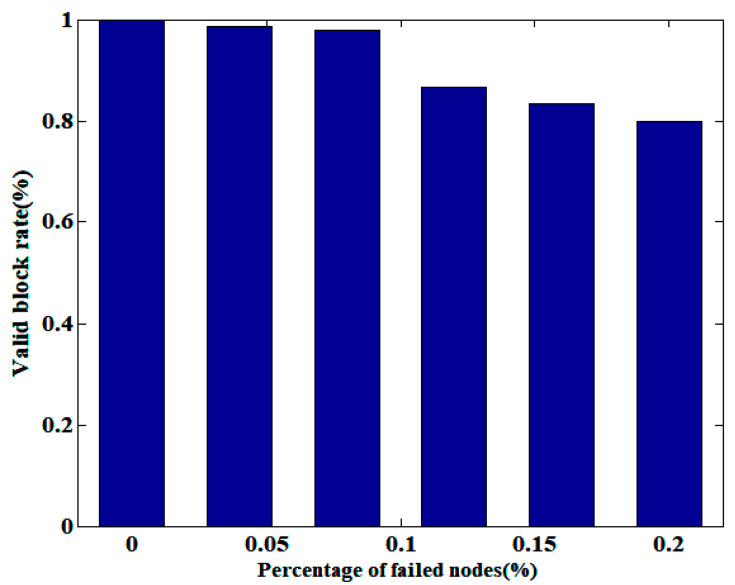
The impact of the percentage of failed nodes on the valid block rate.

The valid block rate is the ratio of the number of blocks successfully appended to the blockchain to the number of blocks generated within a certain period of time. The number of failed nodes is the sum of the number of malicious nodes and the number of nodes whose response time exceeds the set threshold. Figure 8 shows the variation of the valid block rate with an increasing percentage of failed nodes when the block size is 1MB. From this figure, it can be seen that when the percentage of failed nodes is 0, the valid block rate approaches 1, which is due to the presence of a small number of block forks. As the percentage of failed nodes increases, the probability of malicious nodes being selected as the main nodes increases when PBFT is executed in Algorithm 4, resulting in a decrease in the valid block rate. However, the operation of Algorithms 1–3 is independent of the main nodes, and the liveness can also ensure the generation of valid blocks over a period of time. Therefore, the impact of failed nodes on the performance of the proposed CBPA architecture is limited.

By now we have evaluated the performance of the proposed CBPA architecture, such as transaction throughput and transaction latency. We have also shown the impact of node failure on blocking time and valid block rate, indicating the promising performance by the architecture in the IoT. By implementing a node failure recovery scheme [44,45,46], transaction throughput can be further improved and transaction latency can be reduced. However, there is still room for improvement in the real-time transaction requirements, such as emergency transactions or online games, as the transaction throughput does not satisfy the real-time transaction requirements.

### 5.3. Discussion

We perform a sensitivity analysis of CBPA in terms of node dynamics, transaction dynamics, and block size. First, node dynamics refer to the impact of node joining/leaving on the CBPA architecture, and during the experiments, node joining/leaving currently has no impact on block generation. Secondly, for transaction dynamics, the experiments found that when the block size is fixed, there is an upper bound on the transaction throughput, and even if the number of transactions continues to increase, the throughput value does not change significantly. Finally, when the block size continues to increase, the number of transactions that can be accommodated in each block also increases, and theoretically the transaction throughput will also increase. However, in our experiments, it is found that the dynamics of IoT nodes and network latency makes the data transmission time of larger blocks longer. Even the number of packet retransmission becomes more frequent and block transmission failure occurs, which greatly affects the processing time of nodes to obtain the latest block information and reach consensus, and ultimately leads to the existence of an upper bound of transaction throughput. Therefore, the main sensitivity factors affecting CBPA are transaction dynamics and block size.

Although the consensus mechanism like PoW consumes energy, the proposed CBPA can work in the IoT environment for the following three reasons. (1) PoW can effectively prevent Sybil attacks. Compared with PoS and Dpos, the number of communications between nodes is low, which is in line with the actual situation of IoT environment. (2) CBPA divides nodes into two types based on computing power and storage capacity, and they participate in different blockchain activities. (3) The block generator sets the mining difficulty for generating carrier blocks in Section 4.2. When the mining difficulty is too high, it consumes a lot of energy. At the same time, the generation time of the new block is prolonged, and there is a high probability that this block cannot reach consensus in the current epoch. Therefore, when the nodes involved in block generation know the mining difficulty of the carrier block and find that the mining difficulty is too high, they will not participate in the generation of the carrier block.

The main application field of CBPA can be described as nodes executing transactions in a blockchain manner under IoT networks. Similar to nodes under the existing Bitcoin network, nodes under the CBPA architecture are also designed to generate benefits, such as a transaction fee and block generation fee. So, in the CBPA, devices such as sensors, routers, and switches do not participate in blockchain activities, whereas devices with certain computing and storage capacity such as mobile phones, cars, servers, tablets, computers, servers, etc. can participate in blockchain activities in order to gain benefits. Since benefits are a natural incentive, each node rationally calculates the cost–benefit ratio, and compared with PoW in the Bitcoin network, CBPA requires much less computing power from individual nodes. Furthermore, nodes in the CBPA are free to join and leave at any moment because of the blockchain system’s anonymity (which is also consistent with the node dynamics in IoT networks). Meanwhile, as long as the transaction conforms to the blockchain rules, it can be confirmed by the nodes in the CBPA, which has a certain degree of scalability. To sum up, CBPA supports large-scale IoT networks in terms of computational overheads and scalability.

## 6. Conclusions and Future Work

This paper employs a social network graph as a carrier to develop a CBPA architecture for IoT networks. The CBPA architecture includes the generation of carriers, carrier blocks, and transaction blocks, parallel generation of transaction blocks, intra-block and cross-block conflict resolution, and block consensus and fork processing. CBPA enables nodes with high communication latency to obtain legal benefits based on previous contributions as well. Theoretical analysis indicates that CBPA has both security and liveness. The experimental results show that when the block size is 1MB, CBPA improves the average throughput by about 10% and reduces the average latency by about 14% compared to existing schemes. The proposed CBPA architecture expands the applicable blockchain network and provide a feasible scheme for IoT networks.

Despite the proposal of a block parallel generation method in the CBPA architecture, the transaction throughput remains low. This is due to the fact that the proposed method is based on a chain-structured and PoW calculation. In addition, CBPA uses the PoW during block generation, which has good performance in preventing 51% attacks, but is not friendly to IoT devices that employ small-capacity batteries. The subsequent research will be carried out in two aspects: on the one hand, a sharding scheme will be introduced to further improve the parallelism of the nodes participating in the block activity, thus increasing the transaction throughput. On the other hand, alternatives to PoW will be investigated to further expand the number and types of nodes that can participate in the IoT network, thereby reducing energy consumption during block generation and consensus.

## Data Availability

Data used in the experiments were obtained from https://xblock.pro/xblock-eth.html (accessed on 2 August 2024).
